# Comparative evaluation of matrix systems on proximal contact strip force and surface geometry in class II direct composite restorations: an in vitro study

**DOI:** 10.1186/s12903-026-09602-x

**Published:** 2026-08-14

**Authors:** Aqeel A Noorudeen, S Vidhyadhara Shetty, Jeslee Ann Jose, Shyam Ranjan Sharma, Jayaprakash K

**Affiliations:** 1 Department of Conservative Dentistry and Endodontics, Yenepoya Dental College, Yenepoya (Deemed to be University), Mangaluru, Karnataka 575018 India; 2 Department of Dental Materials, Biomaterials and Research Centre, Yenepoya Dental College, Yenepoya (Deemed to be University), Mangaluru, Karnataka 575018 India

**Keywords:** Circumferential matrix system, Class II composite restoration, Proximal contact strip force, Proximal surface geometry, Sectional matrix system

## Abstract

**Background:**

Proximal contact strip force and surface geometry remain challenging in Class II composite restorations, with matrix system selection being a key factor. This study aimed to compare the proximal contact strip force and surface geometry of the myQuickmat Classico sectional matrix system and the myQuickmat all-round circumferential matrix system in Class II MOD composite resin restorations.

**Methods:**

Twenty typodont molar teeth (Nissin Dental Products Inc., Kyoto, Japan) with standardised MOD cavities were selected and equally allocated into two groups. Group 1 consisted of the myQuickmat Classico sectional matrix system (Polydentia SA, Mezzovico-Vira, Switzerland), and Group 2 consisted of the myQuickmat all-round circumferential matrix system (Polydentia SA, Mezzovico-Vira, Switzerland). Composite restorations were done using Tetric N-Ceram composite resin (Ivoclar Vivadent, Liechtenstein). Proximal contact strip force and surface geometry were evaluated using a universal testing machine and confocal profilometer (NANOVEA ST 400, USA).

Normality was confirmed by the Shapiro-Wilk test, and differences between groups were analyzed by Student’s t-test and the Mann-Whitney U test (Jamovi 2.6.44); P < 0.05.

**Results:**

The sectional matrix produced greater proximal contact strip force at mesial (1.903 ± 0.333 N vs. 1.473 ± 0.413 N; P = 0.020) and distal (2.807 ± 0.557 N vs. 1.721 ± 0.405 N; P < 0.001) surfaces. Both systems showed surface concavity; the circumferential system had significantly greater concavity area, depth, and smaller radius of curvature compared with the sectional matrix system (P < 0.001).

**Conclusions:**

The sectional matrix outperformed the circumferential system; however, concavity was observed on the proximal surface with both systems, reflecting limitations in the design of the matrix band.

## Background

The use of direct resin composite materials for class II posterior restorations has increased considerably over the last two decades due to improvements in resin composite material properties, advancements in resin adhesives, and patient demands for tooth-colored posterior restorations. Although posterior resin composite restorations have shown high survival rates, a considerable failure rate still exists. According to Patel P et al. [1] the overall annual failure rate of resin composite restorations was reported as approximately 1–3% in permanent teeth, with a commonly cited mean of 2.8% for posterior restoration. Fracture of the restoration material and defective marginal adaptation contributed to nearly a quarter of the total failures. Santos et al. [2] carried out a five-year retrospective clinical study on posterior resin composite restorations. The results showed that composite restorations scored significantly fewer ideal anatomy scores compared to amalgam restorations (22.4% vs. 49.3%, *p* = 0.0005), thus suggesting that restoration geometry remains a limitation of resin composite restorations.

A physiologically acceptable proximal contact is essential for the success of any class II restoration. Inadequate proximal contacts have been associated with clinical complications such as food impaction, plaque accumulation, secondary caries, and periodontal deterioration in previous clinical studies [3]. The restoration of this contact using composite resin is more difficult compared to amalgam due to the inherent inability of composite resin to be adapted against the matrix and the volumetric shrinkage of 2–4%, which is exacerbated by the high C-factor associated with Class II preparations. The high C-factor is due to the limited stress relief available in Class II preparations, which can cause gaps at the proximal surface [4, 5].

The choice of matrix system appears to be a key technical factor in determining proximal contact results in direct composite restorations. [6, 7] The commonly used circumferential systems, which were originally designed for use in amalgam restorations, have been shown to underperform in composite resin restorations in all situations. Recent systematic reviews have also concluded that sectional matrix systems generally produce superior proximal contact outcomes compared with circumferential systems in Class II composite restorations [8].

A study by Loomans et al. [9] showed a significantly looser proximal contact in circumferential matrices compared to sectional systems in vitro (*p* < 0.001). Clinical results have shown that a satisfactory proximal contact is achieved in only 20% of restorations placed using a Tofflemire system [7]. On the contrary, sectional matrix systems using a mechanical separation ring are considered the gold standard in proximal contact reconstruction in Class II composite resin restorations due to their consistent demonstration of better contact tightness in both in vitro and in vivo studies [10, 11]. This is achieved by a focused lateral force at the contact area provided by the separation ring, which may produce a rebound effect of the adjacent tooth against the restoration after polymerization, thus achieving a better proximal contact than is possible by any circumferential system [5].

Despite their contact tightness advantage, sectional matrices have been shown to produce concave proximal surface contours at the restored contact area — a consequence of band deformation under ring force that creates plaque-retentive zones and compromises interproximal hygiene access. [12].

Although the superiority of sectional matrix systems in achieving proximal contact strip force has been demonstrated in previous studies, [13] most investigations have primarily focused on two-surface Class II restorations or evaluated only proximal contact outcomes. Limited evidence exists regarding their performance in mesio-occluso-distal (MOD) restorations, where simultaneous proximal reconstruction presents additional technical challenges. Furthermore, newer circumferential systems incorporating pre-contoured bands and integrated tightening mechanisms have not been adequately compared with sectional systems in this cavity configuration.

In addition, previous studies have largely focused on contact tightness alone, with comparatively limited evaluation of proximal surface geometry using three-dimensional analytical methods. Since proximal contour morphology may influence plaque retention and interproximal hygiene access, simultaneous assessment of both contact tightness and surface geometry may provide a more comprehensive understanding of matrix system performance.

Therefore, the present study aimed to compare a sectional matrix system with a contemporary circumferential matrix system in standardized Class II MOD restorations using both proximal contact strip force testing and three-dimensional confocal profilometric surface analysis.

Based on previous evidence demonstrating superior proximal contact outcomes with sectional matrix systems, the null hypothesis of the present study was that there would be no significant difference between the sectional and circumferential matrix systems in terms of proximal contact strip force and proximal surface geometry in Class II MOD composite restorations.

## Methods

This in vitro study was approved by the Institutional Ethical committee (Protocol No. YEC2/2024/036). As no human participants or animal subjects were involved, no patient consent was required. The study was conducted in the Department of Conservative Dentistry and Endodontics at a university-affiliated dental institution.

**Fig. 1:**
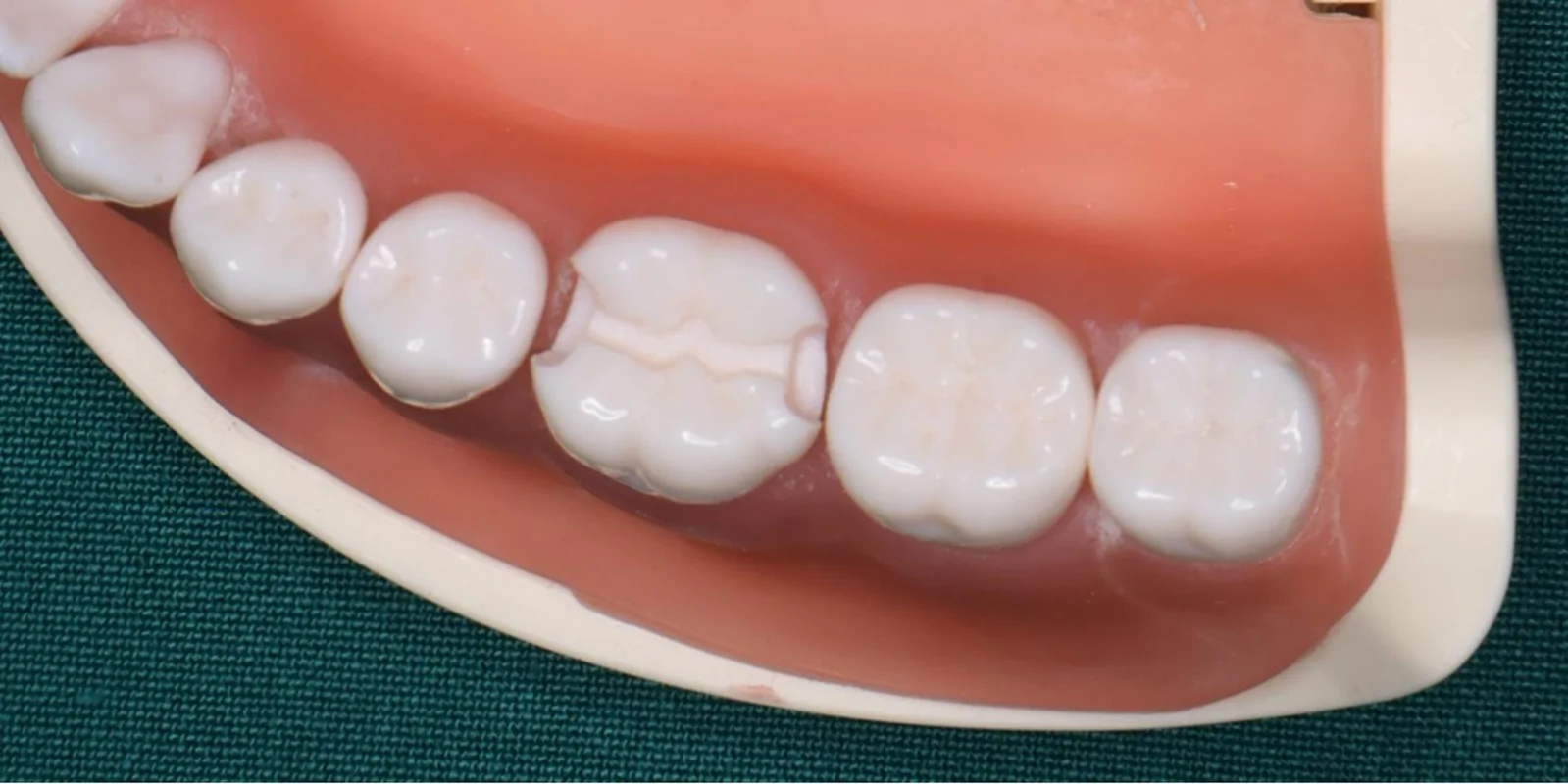
Standardized MOD cavity preparation. Representative typodont mandibular first molar showing the standardized mesio-occluso-distal cavity design used in the study before placement of the matrix systems and composite restorations

The sample size was calculated using G*Power (version 3.1.9.7) with an alpha of 0.05, power of 80%, and an effect size of 1.40 based on a prior study, [5] yielding 20 teeth (ten per group). Twenty typodont lower left first permanent molar teeth (Nissin Dental Products Inc., Kyoto, Japan) were selected to ensure morphological uniformity and minimize biological variability, consistent with prior matrix system investigations [5, 14]' Standardised mesio-occluso-distal (MOD) cavity preparations were performed by a single operator using diamond burs in a high-speed handpiece under water cooling. The MOD cavity preparations were performed with an occlusal isthmus width of 1.5 mm and pulpal depth of 1.5 mm. The proximal boxes were standardised to 4 mm buccolingually, 3 mm occlusogingivally, and 1.5 mm mesiodistally. (Fig. 1) The typodont teeth were retained in their original sockets within the phantom head assembly supplied by the manufacturer. No additional fixation materials or mounting procedures were employed. All cavity preparations, restorative procedures, and measurements were performed with the teeth maintained in this standardized position. Specimens were then randomly allocated into two groups of ten.

Group 1 was restored using the myQuickmat Classico sectional matrix system (Polydentia SA, Mezzovico-Vira, Switzerland) with a mechanical separation ring. Group 2 was restored using the myQuickmat all-round circumferential matrix system (Polydentia SA, Mezzovico-Vira, Switzerland) with an integrated tightening mechanism. Both groups used pre-contoured stainless steel matrix bands with comparable nominal dimensions (approximately 6.5 mm height and 0.03 mm thickness) according to manufacturer specifications. However, the systems differed in matrix configuration and separation mechanics, with the sectional matrix using a separation ring and the circumferential system incorporating an integrated tightening mechanism.

**Fig. 2:**
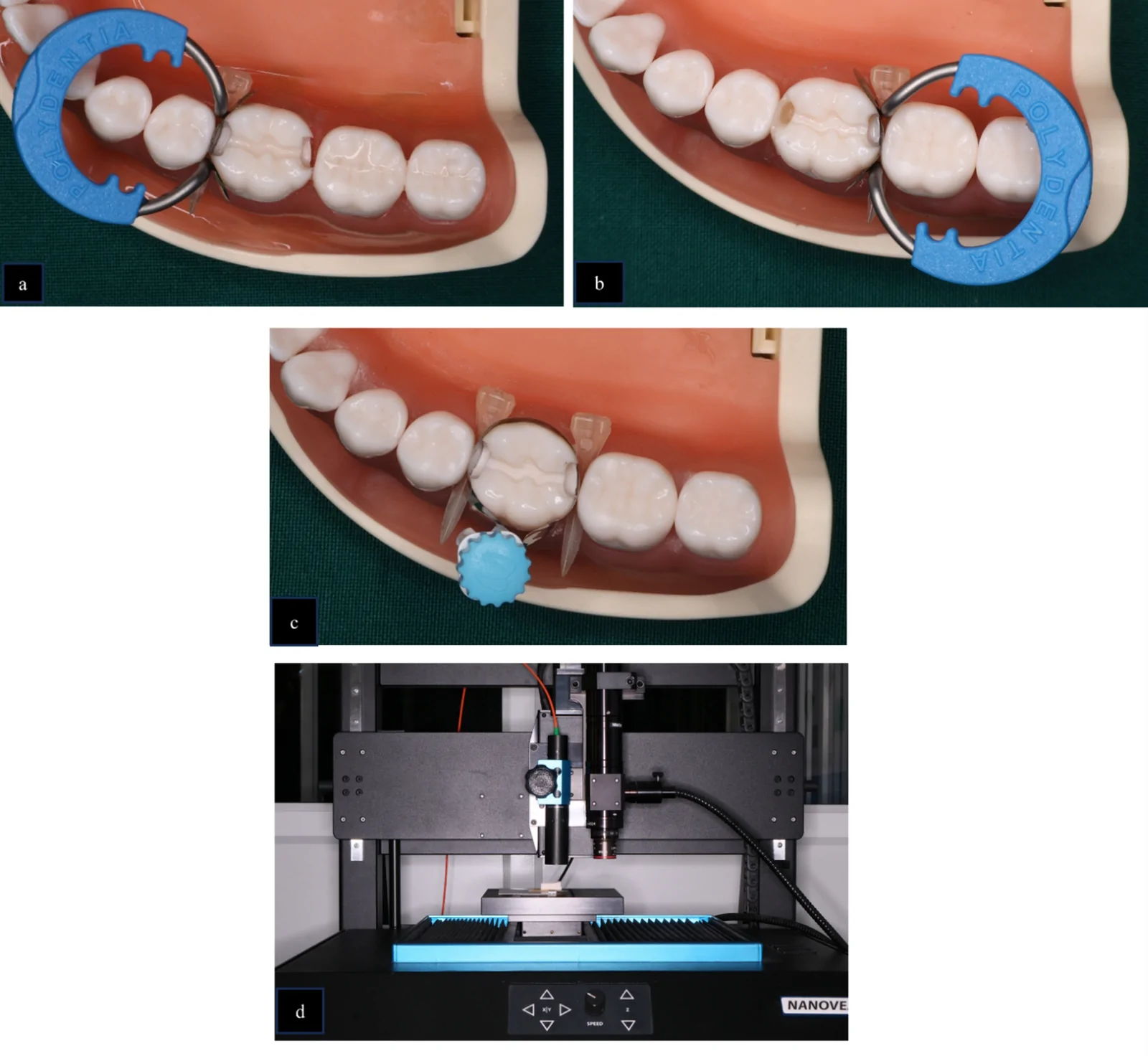
**a** Application of sectional matrix system (myQuickmat Classico, Polydentia) sequentially at the mesial proximal box (Group 1). **b** Sequential application of the sectional matrix system at the distal proximal box (Group 1), following completion of mesial restoration. **c** Application of circumferential matrix system (myQuickmat All-Round, Polydentia) around prepared tooth (Group 2). **d** Evaluation of proximal surface geometry using 3D non-contact confocal profilometer (ST400, NANOVEA, USA)

In Group 1, restoration of the proximal surfaces was performed sequentially, where a sectional band and separation ring were placed on the mesial surface, followed by adaptation and complete polymerization of the composite, after which the assembly was removed and repeated on the distal surface separately [Figure 2a, b]. In Group 2, a circumferential band was placed around the entire tooth and secured with the integrated tightener [Figure 2c]. Anatomical plastic wedges (The Wedge, Polydentia SA, Mezzovico-Vira, Switzerland) were placed gingivally to secure the gingival margin in both groups, and matrix band burnishing was not performed in either group. Tetric N-Bond Universal adhesive (Ivoclar Vivadent, Schaan, Liechtenstein) was applied per manufacturer’s instructions and photo-polymerized for 10 s using a LED curing unit (Woodpecker ILED Plus, Guilin Woodpecker Medical Instrument Co., Ltd., China) at a manufacturer-specified light intensity of 2,300–2,500 mW/cm² within a wavelength spectrum of 420–480 nm, with the light tip held as close to the restoration surface as the cavity access permitted, at a distance of approximately 1 mm. Light intensity was not verified with a radiometer prior to each use. Tetric N-Ceram nano-hybrid composite resin (Ivoclar Vivadent, Schaan, Liechtenstein) was placed in three increments, each cured for 20 s. The first increment was adapted against the matrix band to reconstruct the proximal box and proximal wall, the second increment was placed to complete the remaining proximal and occlusal portions of the cavity, and the third increment restored the final occlusal anatomy. Each increment was individually photo-polymerized for 20 s.

Proximal contact strip force was evaluated using a universal testing machine (Tecsol-TSI-BDS, Tecsol, India). A 0.05 mm stainless steel strip was positioned in the interproximal contact area and withdrawn under tensile loading at a crosshead speed of 5 mm/min. Proximal contact strip force was quantified as the peak frictional force (N) required to withdraw the stainless-steel strip from the contact area, consistent with methods used in previous studies employing the Tooth Pressure Meter technique [13].

Proximal surface geometry was assessed using a three-dimensional non-contact confocal profilometer (ST 400, NANOVEA, Irvine, USA; Expert 3D GP software, version 8.2.10392) [Figure 2d]. Each restored tooth was mounted with the proximal surface perpendicular to the optical axis and scanned in surface topography mode. Following least-squares plane correction of the dataset, concavity area (mm²), depth (mm), and radius of curvature (mm) were computed at both the mesial and distal proximal surfaces using the software’s region-of-interest function.

Data were analysed using Jamovi (version 2.6.44). Normality was assessed using the Shapiro–Wilk test. Normally distributed variables were compared between groups using the independent-samples Student’s *t*-test and reported as mean ± standard deviation. The Radius of Curvature variables, which were not normally distributed, were compared using the Mann–Whitney *U* test and reported as median with interquartile range. A *P* value of < 0.05 was considered statistically significant.

## Results

Detailed results are presented in Tables 1 and 2.

**Table 1:** Comparison of proximal contact strip force between the sectional matrix system (Group 1, myQuickmat Classico, Polydentia SA, Mezzovico-Vira, Switzerland) and the circumferential matrix system (Group 2, myQuickmat all-round, Polydentia SA, Mezzovico-Vira, Switzerland)

Parameter	Group	N	Shapiro–Wilk				*t*-test^†^ / Mann–Whitney U^‡^			
			Mean	Median	SD	IQR	Statistic	*p*-value	Statistic	*p*-value
Mesial PCSF (N)	1	10	1.903	1.825	0.333	0.330	0.881	0.133	2.56^†^	**0.020** ^*^
	2	10	1.473	1.530	0.413	0.565	0.952	0.690		
Distal PCSF (N)	1	10	2.807	2.750	0.557	0.550	0.891	0.175	4.98^†^	**< 0.001** ^*^
	2	10	1.721	1.690	0.405	0.370	0.956	0.745		

^*^ Statistically significant (*p* < 0.05)

^**†**^ Independent-samples Student’s *t*-test

^**‡**^ Mann–Whitney U test; applied where Shapiro–Wilk test indicated non-normality (*p* ≤ 0.05)

PCSF = Proximal contact strip force; SD = Standard Deviation; IQR = Interquartile Range. Group 1: myQuickmat Classico (Polydentia SA, Mezzovico-Vira, Switzerland); Group 2: myQuickmat all-round (Polydentia SA, Mezzovico-Vira, Switzerland)

**Table 2:** Comparison of restoration surface geometry parameters between the sectional matrix system (Group 1, myQuickmat Classico, Polydentia SA, Mezzovico-Vira, Switzerland) and the circumferential matrix system (Group 2, myQuickmat all-round, Polydentia SA, Mezzovico-Vira, Switzerland)

Parameter	Group	N	Shapiro–Wilk				*t*-test^†^ / Mann–Whitney U^‡^			
			Mean	Median	SD	IQR	Statistic	*p*-value	Statistic	*p*-value
Area M (mm²)	1	10	2.446	2.424	0.217	0.258	0.971	0.897	−6.39^†^	**< 0.001** ^*^
	2	10	3.149	3.163	0.272	0.378	0.963	0.817		
Area D (mm²)	1	10	2.351	2.333	0.143	0.238	0.933	0.477	−11.44^†^	**< 0.001** ^*^
	2	10	3.223	3.211	0.194	0.272	0.961	0.800		
Depth M (mm)	1	10	0.157	0.152	0.026	0.035	0.973	0.916	−5.06^†^	**< 0.001** ^*^
	2	10	0.266	0.273	0.063	0.069	0.861	0.078		
Depth D (mm)	1	10	0.153	0.149	0.031	0.045	0.968	0.875	−8.02^†^	**< 0.001** ^*^
	2	10	0.281	0.277	0.039	0.057	0.971	0.900		
Radius of Curvature M (mm)	1	10	2.058	2.197	0.433	0.293	0.768	0.006	3.00^‡^	**< 0.001** ^*^
	2	10	0.905	0.911	0.069	0.088	0.978	0.953		
Radius of Curvature D (mm)	1	10	2.272	2.285	0.156	0.219	0.967	0.866	0.00^‡^	**< 0.001** ^*^
	2	10	0.959	0.857	0.364	0.121	0.546	< 0.001		

^*^ Statistically significant (*p* < 0.05)

^**†**^ Independent-samples Student’s *t*-test

^**‡**^ Mann–Whitney U test; applied where Shapiro–Wilk test indicated non-normality (*p* ≤ 0.05)

Area M/D = Concavity area at mesial/distal surface; Depth M/D = Concavity depth at mesial/distal surface; Radius of Curvature M/D = Radius of curvature of proximal concavity at mesial/distal surface; SD = Standard Deviation; IQR = Interquartile Range; M = Mesial; D = Distal. Group 1: myQuickmat Classico (Polydentia SA, Mezzovico-Vira, Switzerland); Group 2: myQuickmat all-round (Polydentia SA, Mezzovico-Vira, Switzerland)

Group 1 (sectional matrix) produced significantly greater proximal contact strip force than the circumferential matrix group at both mesial and distal surfaces (Table 1).

Within the sectional matrix group, proximal contact strip force was greater at the distal surface compared with the mesial surface. In contrast, the circumferential matrix group demonstrated relatively similar proximal contact strip force values between mesial and distal surfaces. However, statistical comparisons between mesial and distal surfaces within each matrix system were not planned a priori and were not performed; therefore, no conclusions regarding intra-group surface differences can be made.

**Fig. 3:**
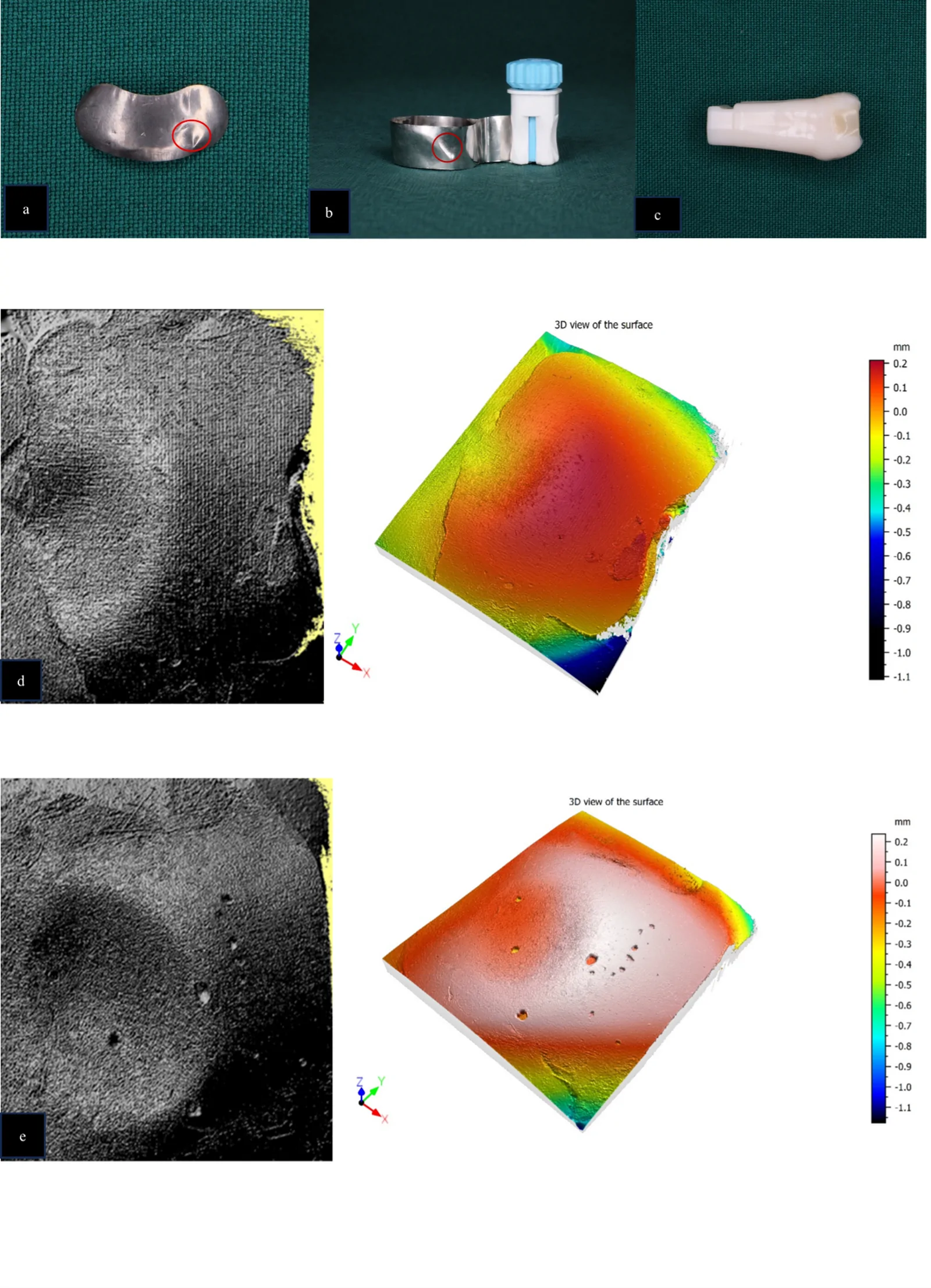
**a** Deformation of sectional matrix band (myQuickmat Classico) after removal. **b** Deformation of circumferential matrix band (myQuickmat all-round) after removal. **c** Proximal surface concavity observed in restored tooth. **d** Three-dimensional confocal profilometry image of proximal surface concavity in restoration completed using the sectional matrix system. **e** Three-dimensional confocal profilometry image of proximal surface concavity in restoration completed using the circumferential matrix system

Proximal surface concavity was observed with both matrix systems. However, the circumferential matrix group demonstrated significantly greater concavity area and depth, along with a significantly smaller radius of curvature, at both mesial and distal surfaces compared with the sectional matrix system (Table 2; Fig. 3a–e).

## Discussion

The present study compared sectional and circumferential matrix systems in Class II MOD composite restorations. Sectional matrices demonstrated superior proximal outcomes. Proximal surface concavity was observed with both systems, although it was significantly greater in the circumferential group. Therefore, the null hypothesis was rejected.

Previous studies have demonstrated superior proximal contact strip force with sectional matrix systems [5, 15, 16]. However, most investigations evaluated only contact tightness on two-surface restorations. The use of confocal profilometry also enabled quantitative assessment of proximal concavity characteristics that are less frequently investigated in previous matrix system studies.

The greater proximal contact strip force observed with the sectional matrix system is consistent with previous in vitro and clinical studies. Previous clinical studies have also reported superior proximal contact outcomes with sectional matrix systems [5, 7, 9, 13]. This advantage is likely related to temporary tooth separation produced by the sectional ring system.

Previous studies have suggested that the advantage of sectional matrix systems may decrease in MOD restorations when two separation rings are used simultaneously [13]. In the present study, this was addressed by restoring the proximal surfaces sequentially, allowing only one ring to be active at a time. This approach may have contributed to the improved contact tightness observed with the sectional system.

An additional methodological consideration is that the sectional matrix restorations were performed sequentially, with the mesial restoration completed and polymerized before restoration of the distal surface [17]. The previously polymerized mesial restoration may have provided additional structural support during distal matrix adaptation and composite placement, potentially influencing force distribution, tooth separation behaviour, and proximal contact formation at the distal surface. This may partly explain the greater distal contact tightness observed in the sectional matrix group and represents a potential source of methodological bias that should be considered when interpreting the findings.

Within the sectional matrix group, the distal surface demonstrated numerically greater proximal contact strip force than the mesial surface, similar to findings reported in previous MOD studies [17]. However, formal statistical comparisons between mesial and distal surfaces within each matrix system were not performed in the present study; therefore, these observations should be interpreted descriptively rather than as statistically confirmed intra-group differences. The observed variation may partly relate to the sequential restoration protocol used in the sectional matrix group, where the previously polymerized mesial restoration could have provided additional structural support and altered matrix adaptation or force transmission during restoration of the distal surface.

Proximal surface concavity was observed with both matrix systems, indicating that this remains a limitation of current matrix band designs. However, the circumferential matrix system produced greater concavity and sharper proximal contours than the sectional system. Similar findings have been reported in previous studies evaluating proximal contour morphology in Class II restorations [12, 18, 19]. This finding may be related to greater matrix band deformation during circumferential tightening. Although both matrix systems used bands with comparable nominal dimensions, minor differences in matrix band design, contour geometry, and stabilization mechanisms may also have influenced proximal contact strip force and surface morphology. Matrix band thickness, height, pre-contouring characteristics, and resistance to deformation may affect matrix adaptation during composite placement and polymerization. Therefore, the observed differences should not be interpreted as being solely attributable to the separation mechanism of the matrix systems.

Clinically, inadequate proximal contacts and improper proximal contours have been associated with food impaction and plaque retention, which may increase the risk of secondary caries and periodontal complications [3]. Previous clinical investigations have reported superior proximal contact outcomes with sectional matrix systems [15, 20]. However, the present study was conducted under in vitro conditions; therefore, the findings should be interpreted as laboratory observations that may not directly predict long-term clinical outcomes.

Although statistically significant differences in proximal contact strip force were observed between the matrix systems, the clinical relevance of these differences should be interpreted cautiously. Previous studies have suggested that excessively loose proximal contacts may contribute to food impaction and plaque accumulation, [3, 15], whereas overly tight contacts may complicate floss passage and patient comfort [13, 21]. In the present study, the sectional matrix system produced consistently tighter contacts than the circumferential system; however, the extent to which the observed differences exceed clinically meaningful thresholds remains unclear because universally accepted quantitative standards for optimal proximal contact strip force have not yet been established [13, 21, 22]. Therefore, the findings should primarily be interpreted as evidence of relative mechanical superiority under controlled laboratory conditions rather than direct predictors of long-term clinical success.

A major strength of the present study was the simultaneous evaluation of proximal contact strip force and three-dimensional proximal surface geometry in standardized MOD restorations, allowing assessment of both functional contact integrity and contour morphology. In addition, the comparison involved a contemporary circumferential matrix system with an integrated tightening mechanism rather than a conventional Tofflemire system, improving the clinical relevance of the findings.

This study has several limitations. Although typodont teeth improved standardization, they do not reproduce the biomechanical behavior of natural teeth or periodontal ligament compliance. In addition, the in vitro design could not simulate important intraoral conditions such as saliva, thermal fluctuations, and functional occlusal loading. The sample size was relatively small (*n* = 10 per group); although determined using an a priori power analysis, it may still limit the generalizability of highly variable three-dimensional geometric measurements. Operator and assessor blinding were not performed due to the nature of the experimental procedures, which may represent a potential source of measurement bias despite the use of objective instrument-based outcome measurements. Furthermore, only one tooth type and cavity design were evaluated, and thermocycling and mechanical fatigue loading were not performed. Therefore, the findings represent immediate post-restoration laboratory outcomes and should be interpreted cautiously when extrapolating to long-term clinical performance.

Future clinical studies are needed to determine whether the improved proximal contact strip force and surface geometry observed in this laboratory model translate into meaningful long-term clinical benefits.

## Conclusions

Within the limitations of this in vitro study, the sectional matrix system with a mechanical separation ring (myQuickmat Classico, Polydentia SA, Mezzovico-Vira, Switzerland) demonstrated greater proximal contact strip force and more favourable proximal surface geometry compared with the circumferential matrix system with an integrated tightening mechanism (myQuickmat all-round, Polydentia SA, Mezzovico-Vira, Switzerland) in Class II MOD direct composite restorations. Proximal surface concavity was observed with both systems, suggesting persistent limitations in current matrix band designs. These findings suggest that matrix separation mechanics appear to influence proximal contact strip force and contour morphology; however, the clinical relevance of the observed differences in proximal contact strip force and surface geometry remains to be clarified, as universally accepted quantitative thresholds for these parameters have not yet been established, and long-term clinical studies are warranted. The present study also contributes additional evidence regarding proximal surface contour alterations associated with different matrix separation mechanisms in MOD restorations.

## Data Availability

The raw data supporting the conclusions of this article are not publicly deposited in a repository but are available from the corresponding author upon reasonable request.

## Abbreviations

MOD: Mesio-occluso-distal
PCSF: Proximal contact strip force
SD: Standard Deviation
IQR: Interquartile Range
LED: Light-Emitting Diode
3D: Three-dimensional
